# HIV-Indicator Condition Guided Testing in a Hospital Setting

**DOI:** 10.3390/life13041014

**Published:** 2023-04-14

**Authors:** Diletta Barbanotti, Camilla Tincati, Alessandro Tavelli, Andrea Santoro, Matteo Sala, Teresa Bini, Anna De Bona, Antonella d’Arminio Monforte, Giulia Carla Marchetti

**Affiliations:** Unit of Infectious Diseases, ASST Santi Paolo e Carlo, Department of Health Sciences, University of Milan, 20122 Milan, Italy

**Keywords:** HIV, AIDS, screening, late diagnosis, HIV indicator conditions

## Abstract

Late diagnosis is still a major issue in HIV infection management, leading to important consequences for both patients and community. In this perspective, HIV screening targeted on some clinical conditions (HIV indicator conditions—HIVICs) emerged as a useful strategy, also involving patients not considered at high behavioral risk. We organized an in-hospital HIVICs guided screening campaign named ICEBERG in Milan, Italy, between 2019 and 2021. Among the 520 subjects enrolled, mainly presenting with viral hepatitis or mononucleosis-like syndrome, 20 resulted HIV positive (3.8% prevalence). A significant proportion of them had multiple conditions and advanced immunosuppression, with 40% being AIDS-presenters. As adherence to the screening campaign was modest for non-ID specialists, educational interventions to raise clinicians’ sensitivity are urgently needed. HIV-ICs guided testing was confirmed as a useful tool, but a combined approach with other screening strategies seems to be essential for early HIV diagnosis.

## 1. Introduction

Despite enormous progresses in HIV infection management achieved in the last 25 years, UNAIDS 2020 90-90-90 targets (90% of people living with HIV being diagnosed, 90% of them being on treatment, 90% of them being virologically suppressed) were not universally achieved [1,2].

While in many countries antiretroviral (ARV) treatment availability is still limited, thus hindering the achievement of the second and third UNAIDS 90 targets, in Western Europe, availability of ARV drugs is widespread while the first target seems to be the hardest to reach. In fact, according to epidemiological European reports, up to 1 out of 7 people living with HIV (PLWH), estimated in 120,000 individuals, are not aware of their status [3]. Moreover, almost half of those newly diagnosed has advanced immunosuppression at the time of diagnosis, defined as ‘late presenters’ or ‘late diagnosed’, with CD4+ lymphocytes count less than 350/mm^3^. Most of them had previously sought medical attention on one or more occasion for conditions that should have prompted HIV testing [3,4]. This delay in diagnosis, estimated in up to three years, can lead to high morbidity and mortality as immunodeficiency develops, increased healthcare-related costs and, moreover, onward transmission of infection [5,6,7,8].

Therefore, screening strategies and early diagnosis achievement have a major role in the effort to control and eventually stop HIV/AIDS epidemic. Since 2008, HIV indicator condition (HIV-IC) guided screening emerged as an alternative to universal and risk behavior driven testing, that showed suboptimal performances. HIV-ICs are clinical conditions more frequently observed in HIV subjects than in seronegative individuals, and their recognition should elicit HIV testing offer [9]. Sullivan, Raben, and colleagues identified in HIDES I and II studies several conditions associated with considerable prevalence of HIV across Europe, with moderate immunodeficiency at diagnosis [10,11]. Screening in settings with HIV prevalence above 0.1% was shown to be cost effective in previous analyses [12]. This strategy showed promising results and therefore was endorsed and encouraged by ECDC, who developed a specific guidance in 2014 [13,14]. Briefly, ICs could be divided into several groups: (I) those resulted from high risk behavior (such as viral hepatitis and other sexually transmitted infections); (II) conditions occurring in subjects with mild immunodepression (such as Herpes zoster infection in people younger than 60 years, or bacterial pneumonia below the age of 50); (III) AIDS defining conditions, and finally, (IV) conditions requiring immunosuppressive treatment (such as chemotherapy for cancer) in which subjects need to be aware of a undiagnosed HIV infection.

Despite some differences in conditions under study, HIV-ICs guided testing is currently recommended by most international and national guidelines, especially in settings with low HIV prevalence [15,16,17].

Italian epidemiological reports match European data, with almost 60% of newly diagnosed patients presenting with advanced immunosuppression, and more than half of them being AIDS-presenters [18].

Specific informed consent for HIV testing requested by Italian legislation appears as a further obstacle to early diagnosis, sometimes hampering serological screening as routine test and posing HIV on another level than other clinical conditions, contributing to stigma [18].

The primary aim of the study was to verify feasibility and effectiveness of an in-hospital condition-guided HIV screening in terms of number of new HIV cases and immune competence upon diagnosis, evaluating its utility in early HIV diagnosis. A secondary objective was to estimate prevalence of previously undiagnosed HIV within each indicator condition.

## 2. Materials and Methods

A HIV-IC guided screening campaign named ICEBERG (HIV sCreening tEst BEyond the taRGet) was organized in ASST Santi Paolo e Carlo, Milan, Italy, from January 2019 to December 2021.

Infectious diseases, Dermatology, Hematology, Oncology, Neurology, Gynecology, Gastroenterology wards, and the Intensive Care Unit were involved, since HIV-ICs were expected to be most frequently observed in such units. Each unit provided a dedicated health care professional, in charge of patients’ enrollment, informed consent obtaining, and HIV test prescription. The study was approved by the local Ethical Committee.

As in HIV in Europe Guidance, HIV-ICs included AIDS-defining diseases, conditions that may indicate initial immunological deficit (herpes zoster in subjects <60 yo, persistent herpes simplex, pneumonia in subjects < 50 yo, mononucleosis-like syndrome, invasive pneumococcal disease in subjects <50 yo, candidemia, dementia in subjects <60 yo, cerebral lesions, persistent leukopenia, and persistent thrombocytopenia), conditions sharing same transmission route with HIV (gonorrhea; viral hepatitis A, B, or C; and syphilis), and those in which an undiagnosed HIV infection could cause severe clinical consequences, such as neoplasm requiring chemotherapy (anal carcinoma, Hodgkin’s lymphoma) [13].

Basic demographic variables including sex, age, ethnicity, and nationality besides one or more HIV-ICs, were collected in an anonymous electronic database. For subjects who resulted HIV positive, basal HIVRNA, CD4+ lymphocytes count, and most plausible transmission modality were investigated.

According to Italian legislation, informed consent was obtained with opt-in modality. Fourth generation antibody-antigen combined whole blood HIV test was employed as screening assay. In case of reactivity, a second sample was collected to perform confirmatory test and Western Blot.

Statistical analysis was performed with Stata (StataCorp (College Station, TX, USA), 2021). Distribution of HIV positivity by demographic variables and HIV-ICs was analyzed by Chi square test and Mann–Whitney U test with 95% confidence intervals. Factors associated with HIV positive test were assessed by uni-and multi-variate logistic regression analysis.

## 3. Results

From January 2019 to December 2021 520 patients presenting with a total of 628 HIV-ICs were enrolled in the study. Most of the population was composed of Caucasian males, mostly Italian, with median age 44 (IQR 32–59) years. However, a substantial rate of foreign patients (*n* = 170, 33%) and non-Caucasian ethnicities (*n* = 134, 26%) were also included.

Most frequently encountered HIV-ICs were viral hepatitis, especially HBV (*n* = 155; 25%), mononucleosis-like syndrome (*n* = 101; 16%), and syphilis, both early and latent (*n* = 72; 11.5%). Among the total cohort, 96 (19%) subjects were suffering from at least one AIDS-defining disease; in detail: 66 had tuberculosis, 2 disseminated CMV infections, 9 non-Hodgkin’s lymphomas, 5 recurring pneumonia, 3 *P. jirovecii* pneumonia, 5 esophageal candidiasis, 6 had dementia, and 1 Kaposi’s sarcoma.

Among the 425 patients presenting with single HIV-IC, 208 (49%) were diagnosed with a sexually transmitted infection, 148 (35%) suffered from a condition possibly related to mild immunosuppression, 63 (15%) had an AIDS-defining disease, and 6 (1%) performed HIV test before starting anticancer treatment (Table 1).

A total of 95 subjects (18%) presented with multiple conditions, with 82 (16% out of total population) of them presenting with 2 HIV-ICs, and the remaining 13 patients with three.

Subjects presenting with an STI, or multiple conditions were frequently males, while the highest proportion of foreign patients was observed among those with AIDS-defining diseases or multiple conditions (Table 1).

A total of 93 out of the 95 subjects with more than 1 condition had at least 1 STI (Table 2). Almost half of this subgroup (41, 43%) presented with multiple STIs, with high prevalence of Italian individuals. By contrast, frequency of non-Italian nationality was highest among subjects presenting with AIDS defining disease and an STI.

### 3.1. New HIV Diagnoses

Twenty new HIV infections were diagnosed, indicating 3.8% (95%CI 2.4–5.9) prevalence. These patients were mainly men, Italian, with median age 39 (32–44) years (Table 3). Most frequently reported transmission route was heterosexual intercourse, accounting for 53%. Among these subjects, 9 (45%) presented with multiple HIV-ICs (Table 4). All these 9 patients were male and had at least 1 STI and another condition, with mononucleosis-like syndrome being the most common.

An amount of 15 patients (80%) were late presenters, with LTCD4 count below 350/mm^3^ or AIDS defining disease, and among them 11 had LTCD4 count below 200/mm^3^, thus defined as ‘with advanced disease’, so that median LTCD4 count was 49/mm^3^; immunological markers were apparently worse among patients with multiple conditions or AIDS defining disease.

### 3.2. Factors Associated with HIV Infection

Sex, nationality, ethnicity, and age did not emerge as significantly associated with HIV test positivity by Chi square or Mann–Whitney U test, although a trend towards younger median age in HIV positive subjects was observed. Moreover, as expected, patients presenting with multiple HIV-ICs resulted more commonly HIV positive (Table 5). Both univariate and multivariate logistic regression analysis confirmed these results, with a moderate risk reduction for 10 years age increment and substantial augmentation for subjects presenting with three conditions (Table 6).

### 3.3. HIV Prevalence within HIV-Ics

HIV infection prevalence resulted above 0.1% in thirteen conditions (esophageal candidiasis, dementia in patients < 60 yo, gonorrhea, herpes zoster in patients < 60 yo, HBV or HCV infection, tuberculosis, disseminated CMV infection, pneumonia in patients < 50 yo, *P. jirovecii* pneumonia, Kaposi’s sarcoma, syphilis, and mononucleosis-like syndrome; Table 7). No HIV infection was detected in nine conditions, all of them were infrequently encountered. Another condition, HAV infection, was quite common in our series, but none among the 52 tested subjects resulted HIV positive.

## 4. Discussion

The present analysis revealed high HIV prevalence in the subset of enrolled hospitalized patients presenting with various clinical conditions, possibly indicating an underlying immunosuppression in Milan, Italy. Condition-guided screening emerged in recent decades as a promising strategy to identify undiagnosed HIV infection, entailing some advantages, that were confirmed in our analysis.

First, HIV test offer is independent from perceived a priori risk. In fact, many studies highlighted how healthcare professionals might not detect some risky exposures, or patients could also not disclose certain behaviors, mainly for the fear of being judged [19,20].

Additionally, several other papers reported that one of the most significant barriers to HIV test execution is a certain degree of hesitation about its offer, mainly because healthcare personnel declare lack of specific training [21,22]. In this perspective, a standardized test offer based on prespecified objective triggers as HIV-ICs can overcome such obstacles and normalize HIV screening as part as routine analysis in presence of some medical conditions. This emerged in our study, as some of the HIV positive patients would not have been identified by risk assessment only, not belonging to high-risk groups.

On the other hand, condition-based strategy allowed to identify a relatively high number of HIV positive subjects by running a limited number of tests, as opposed to universal screening, entailing high volume of test performed to find one case, especially in limited prevalence settings as Wester Europe [23]. Nevertheless, we must consider that this approach results in the identification of possibly long-lasting HIV infection, as LTCD4 cell counts at HIV diagnosis are indicative of late presentation or advanced disease strata. Thus, such an approach cannot be considered the most convenient in terms of public health, as these newly identified PLWH might have transmitted the infection over years being unaware of their condition. Of note, testing these individuals during one of their previous medical encounters might have led to an earlier diagnosis.

Regarding the twenty HIV positive subjects identified in our analysis, their demographic characteristics match those of the newly diagnosed patients according to Italian and European data, with young men under the age of 40 years being the most affected group, and 20–30% originating from high prevalence countries [3,18].

The most striking difference resides in transmission mode: in our analysis male-to-male sexual transmission accounted only for 30%, well below epidemiological data from Italian Registry. Given the limited number of HIV positive subjects in the present study, with transmission mode being declared only for 17 of them, small variations in frequencies reflected consistent fluctuation of relative weight. Moreover, presence of risky behavior was only assessed for the HIV positive subgroup, so that we cannot exclude that this difference may be due to a general limited representation of MSMs in our cohort.

Two main other observations arise from our analysis. First, the great part of the newly diagnosed patients had consistent CD4 lymphocytes depletion, implicating substantial immunosuppression, and 40% had an AIDS-defining condition. These results match data from epidemiological reports, indicating that HIV infection is mostly diagnosed in late stages, with well-known relevant clinical, economic, and public health implications [3]. Condition-based screening seems not to be able to hinder this trend; however, the current study design lacks data on the previous medical encounters of newly diagnosed HIV individuals, which may show that indicator-guided testing may be beneficial in identifying HIV in the early stages.

Second, mononucleosis-like syndrome emerged as the most common condition among those subjects (40%), and HIV prevalence among all patients affected by this condition was around 8%. Despite this clinical presentation is quite non-specific and common among the general population, it might hide both an acute retroviral syndrome and a long-course HIV infection revealing with constitutional symptoms. Similar results were presented in HIDES I and II studies, where HIV prevalence among subjects with mononucleosis-like syndrome was 4–5% [10,11]. Based on those results, HIV screening is nowadays recommended in such patients by national and international guidelines [16,24].

Indeed, these symptoms should prompt HIV testing in primary care facilities and/or community pharmacies in which medical staff are likely to see many cases of febrile illnesses, thus potentially increasing the rate of HIV diagnosis in the earliest stages of disease.

In a recent study also investigating feasibility of HIV-ICs guided screening in a Sardinian hospital, De Vito et al. found similar results, with 3.7% prevalence of previously unknown HIV infection. Eight out of eleven HIV positive patients had advanced immunosuppression, and five of them had an AIDS-defining condition at diagnosis [25].

Two-thirds of the study period coincided with the COVID19 pandemics. The complete reorganization of healthcare systems negatively affected all screening campaigns and all activities non-SARS-CoV-2 related [26,27]. Anyway, this could only partially explain the general low adherence to this condition guided screening strategy, as enrollment was limited also in 2019, in the pre-pandemic setting. Previous papers reported poor adherence to HIV testing, both with universal and targeted strategies [19,23,28]. In particular, the same authors of the HIDES studies found low actuation of the strategy they proposed, despite provided evidence of its effectiveness, acknowledged at the international level [29,30].

In our cohort, the great majority of HIV tests were prescribed by either infectious disease specialists or by dermatologists, underlying a certain reluctance in test offer by other clinicians, not routinely involved in HIV and STI management. This could be due to a suboptimal knowledge of international recommendations for HIV screening, and of the prevalence of HIV in the disease they are expert of, but also a lack of confidence in facing such topic, commonly considered limited to some specific populations, actually contributing to HIV-related stigma. On the other hand, a British study reported how in 2017 HIV screening was not mentioned in many guidelines regarding HIV-IC management [31]. In this perspective, guidelines update associated with direct education in terms of HIV prevalence among HIV-ICs and condition guided screening performance could definitely increase clinicians’ awareness towards this subject. Recently, Garcia-Garcia and colleagues showed the effectiveness of a brief educational intervention on non-HIV specialists at a tertiary hospital in Spain. In addition to increasing physicians’ awareness of current guidelines on HIV screening, the authors observed a significant increase in number of prescribed tests and new diagnoses [32].

A possible further deterrent to HIV screening might have been the need for explicit informed consent and pre -test counselling, as required by Italian legislation, being a time-consuming step. This approach adopted in the pre-ART era, sometimes termed HIV-exceptionalism, was overcome in some countries such as the USA with an opt-out strategy, by which patients get tested unless actively declining [33].

Many conditions were extremely rare in our analysis. While some of them may have been rarely observed in fact, some others, such as lymphomas, were presumably much more common than reported and likely triggered HIV test, as part of well-established clinical practice, but were not enrolled in Iceberg study for unknown reasons.

Our analysis has some relevant limitations. First, no retrospective phase was designed, so that it is not possible to detect any difference in terms of HIV test coverage with respect to previous standard.

Moreover, since the total number of patients with HIV-ICs and patients test refusal were not evaluated, no conclusion can be drawn about test offer nor about the gap between subjects who accepted the screening and those enrolled in the study, and selection bias cannot therefore be excluded. Third, the reasons for previous medical contact in newly diagnosed HIV-infected subjects are unavailable; a detailed analysis of these data may lead to findings in contrast to those we report in the present paper by showing that indicator condition-based testing, if broadly and consistently applied, may be extremely useful in identifying people early in the course of HIV infection.

## 5. Conclusions

In conclusion, condition-based HIV screening confirmed to be feasible and highlighted a significant prevalence of newly diagnosed infection in healthcare settings, although immunodepression was already advanced in most cases. However, this strategy remains poorly applied by non-ID clinicians, and its implementation is key to identifying the hidden part of HIV infection. A combined approach, including out-of-hospital screening strategies involving asymptomatic subjects, or those with non-specific symptoms (i.e., mononucleosis-like illness) in primary care facilities/community pharmacies together with changes in legislative aspects, such as opt-out modality testing and educational campaigns among non-ID clinicians, seems to be necessary for early diagnosis, achieving the first UNAIDS 95 goal.

## Figures and Tables

**Table 1:** Demographic features of the 520 included subjects, stratified by number and type of HIV-IC.

		Single HIV-IC *N* = 425 (82%)				Multiple HIV-IC *N* = 95 (18%)	Total Study Population *N* = 520 (100%)
		AIDS-Defining Disease *N* = 63 (12%)	Mild Immunosuppression Related Condition *N* = 148 (28%)	STI *N* = 208 (40%)	Neoplasm *N* = 6 (1%)		
Gender	F	21 (33%)	55 (37%)	45 (22%)	2 (33%)	24 (25%)	147 (28%)
	M	42 (67%)	93 (63%)	163 (78%)	4 (67%)	71 (75%)	373 (72%)
Italian nationality		26 (60%)	114 (77%)	148 (71%)	4 (67%)	58 (61%)	350 (67%)
Ethnicity	Caucasian	28 (65%)	118 (80%)	168 (81%)	5 (83%)	67 (69%)	386 (75%)
	African	4 (6%)	5 (3%)	12 (6%)	0	6 (6%)	27 (5%)
	Hispanic	8 (13%)	8 (5%)	5 (2%)	1 (17%)	7 (7%)	29 (5%)
	Asian	10 (16%)	7 (5%)	7 (3%)	0	11 (11%)	35 (7%)
	Maghrebi	13 (20%)	10 (7%)	16 (8%)	0	4 (4%)	43 (8%)
Age, median (IQR)		47 (34–56)	38 (27–46)	51 (35–67)	57 (36–81)	50 (37–64)	44 (32–59)

**Table 2:** Demographic features of the 95 subjects presenting with multiple HIV-ICs, stratified by type of HIV-IC.

		Multiple HIV-ICs, N = 95					
		Multiple STIs *N* = 41 (43%)	AIDS-Defining Disease + STI *N* = 20 (22%)	Mild Immunosuppression Related Condition + STI *N* = 25 (26%)	STI + Neoplasm *N* = 4 (4%)	AIDS-Defining Disease + Neoplasm *N* = 2 (2%)	AIDS-Defining Disease + Mild Immunodepression-Related Condition + STI *N* = 3 (3%)
Gender	F	10 (24%)	3 (15%)	9 (36%)	1 (25%)	1 (50%)	0
	M	31 (76%)	17 (85%)	16 (64%)	3 (75%)	1 (50%)	3 (100%)
Italian nationality		34 (83%)	5 (25%)	13 (52%)	2 (50%)	2 (100%)	2 (67%)
Ethnicity	Caucasian	36 (88%)	7 (35%)	19 (76%)	2 (50%)	2 (100%)	2 (67%)
	African	0	4 (20%)	1 (4%)	0	0	1 (33%)
	Hispanic	3 (8%)	2 (10%)	2 (8%)	0	0	0
	Asian	1 (2%)	7 (35%)	1 (4%)	1 (25%)	0	0
	Maghrebi	1 (2%)	0	2 (8%)	1 (25%)	0	0
Age, median (IQR)		51 (41–68)	49 (32–58)	42 (28–54)	64 (41–86)	49 (39–58)	52 (40–66)

**Table 3:** Newly diagnosed HIV infections.

Patient	Age	Gender	Nationality	HIV Indicator Conditions	Transmission Route	CD4 Cells/mm^3^	CD4%	HIVRNA Copies/mL
1	32	F	Nigeria	Pneumonia (<50 yo)	Heterosexual	176	9	24.703
2	47	M	Italy	*P. jirovecii* pneumonia	Heterosexual	1	5	16.605
3	23	M	Morocco	Pneumonia (<50 yo); HBV and HCV infection	IDU	1100	25	5.472
4	45	F	Ethiopia	Dementia (<60 yo)	Heterosexual	31	5	130.721
5	40	M	Italy	Gonorrhea; Syphilis	n.a.	n.a.	n.a.	n.a.
6	36	M	Perù	Mononucleosis-like syndrome (acute HIV infection)	MSM	119	5	13,000.000
7	42	M	Italy	HBV infection	IDU	21	8	1146.543
8	54	F	Italy	Herpes zoster (<60 yo)	Heterosexual	224	20	1.333
9	34	F	Italy	Mononucleosis-like syndrome	Heterosexual	27	8	320.323
10	42	M	Italy	Mononucleosis-like syndrome; HCV infection; Syphilis	n.a.	117	16	186.988
11	67	M	Italy	Esophageal candidiasis; HBV infection; Syphilis	Heterosexual	18	1	160.831
12	40	M	Italy	HBV infection; Kaposi’s sarcoma; Mononucleosis-like syndrome	MSM	49	4	167.638
13	32	F	Italy	HBV infection (pregnant patient)	Heterosexual	361	29	25.329
14	32	M	Italy	Mononucleosis-like syndrome	MSM	6	3	69.517
15	27	M	Italy	Mononucleosis-like syndrome	n.a.	1706	34	544.599
16	51	M	Italy	Esophageal candidiasis; HCV infection; *P. jirovecii* pneumonia	IDU	3	1	39.759
17	24	M	Italy	Mononucleosis-like syndrome; Syphilis	MSM	367	15	4607.623
18	40	M	Sierra Leone	Tuberculosis; HBV infection	Heterosexual	202	18	4047.851
19	31	M	Perù	Syphilis; Mononucleosis-like syndrome; disseminated CMV infection	MSM	19	4	398.424
20	31	M	Italy	*P. jirovecii* pneumonia	Heterosexual	11	2	118.494

**Table 4:** Demographic features and immunological profile of the 20 HIV positive subjects, according to number and type of HIV-IC. “*” indicate that those data are not median but means, as only two patients belong to “STI” cathegory.

		Single HIV-IC and HIV Positive *N* = 11 (55%)			Multiple HIV-ICs and HIV Positive *N* = 9 (45%)	Total HIV-Positive *N* = 20 (100%)
		AIDS-Defining Disease *N* = 3 (15%)	Mild Immunosuppression Related Condition *N* = 6 (30%)	STI *N* = 2 (10%)		
Gender	F	1 (33%)	3 (50%)	1 (50%)	0	5 (25%)
	M	2 (67%)	3 (50%)	1 (50%)	9 (100%)	15 (75%)
Italian nationality		2 (67%)	4 (66%)	2 (100%)	6 (67%)	14 (70%)
Ethnicity	Caucasian	2 (67%)	4 (66%)	2 (100%)	6 (67%)	14 (70%)
	African	1 (33%)	1 (17%)	0	1 (11%)	3 (15%)
	Hispanic	0	1 (17%)	0	1 (11%)	2 (10%)
	Asian	0	0	0	0	0
	Maghrebi	0	0	0	1 (11%)	1 (5%)
Age, median (IQR)		46 (31–48)	34 (31–41)	37 *	40 (29–47)	39 (32–42)
LTCD4/mm^3^, median (IQR)		11 (1–31)	148 (22–595)	941 *	83 (18–326)	49 (11–202)

**Table 5:** HIV infection distribution according to demographic variables by Chi square and Mann–Whitney U test.

		HIV Neg *N* = 500		HIV Pos *N* = 20		
		*N*	Prevalence (CI 95%)	*N*	Prevalence (CI 95%)	*p*
Gender	Female	142/147	97 (92–99)	5/147	3 (1–8)	0.741
	Male	358/373	96 (93–98)	15/373	4 (2–7)	
Italian	No	164/170	97 (92–99)	6/170	3 (1–8)	0.794
	Yes	336/350	96 (93–98)	14/350	4 (2–7)	
Ethnicity	Caucasian	371/385	96 (94–98)	14/385	4 (2–6)	0.155
	African	23/26	89 (70–97)	3/26	11 (3–30)	
	Hispanic	26/28	93 (76–99)	2/28	7 (1–24)	
	Asian	35/35	100 (88–100)	0	-	
	Maghrebi	42/43	98 (87–100)	1/43	2 (0–13)	
Age, median (IQR)		45 (32–59)		39 (32–44)		0.054

**Table 6:** Factors associated with HIV positivity by univariate and multivariate logistic regression analysis.

		Univariate Analysis		Multivariate Analysis	
		OR	*p*	aOR	*p*
HIV-ICs number (vs. 1)	2	1.4 (0.4–5.2)	0.590	1.9 (0.5–7.2)	0.359
	3	32.3 (9.3–111.9)	<0.001	51.5 (12.2–217.3)	<0.001
Male gender (vs. female)		1.2 (0.4–3.3)	0.741	0.8 (0.2–2.4)	0.635
Ethnicity (vs. Caucasian)	African	3.1 (0.8–11.6)	0.087	2.6 (0.6–11)	0.190
	Hispanic	1.9 (0.4–9)	0.396	1.4 (0.3–3.5)	0.710
	Asian	-	-	-	-
	Maghrebi	0.6 (0.1–5)	0.670	0.3 (0.03–3.5)	0.355
Age (10 years increment)		0.7 (0.6–1)	0.051	0.7 (0.5–0.9)	0.015

**Table 7:** HIV infection prevalence within each HIV-IC.

	HIV Neg		HIV Pos	
	*N*	Prevalence (95% CI)	*N*	Prevalence (95% CI)
Candidaemia	2/2	100 (29–100)	0	-
Esophageal candidiasis	3/5	60 (23–88)	2/5	40 (12–77)
Anal carcinoma	1/1	100 (17–100)	0	-
Cervical carcinoma	1/1	100 (17–100)	0	-
Dementia (<60 yo)	5/6	83 (42–99)	1/6	17 (1–58)
Gonorrhea	9/10	90 (57–100)	1/10	10 (0–43)
Herpes zoster (<60 yo)	4/5	80 (36–98)	1/5	20 (2–64)
HAV infection	52/52	100 (92–100)	0	-
HBV infection	149/155	96 (92–98)	6/155	4 (2–8)
HCV infection	57/60	95 (86–99)	3/60	5 (1–14)
Tuberculosis	65/66	99 (91–100)	1/66	2 (0–8)
Atypical micobacteriosis	1/1	100 (17–100)	0	-
Cerebral lesions	5/5	100 (51–100)	0	-
Hodgkin’s lymphoma	2/2	100 (29–100)	0	-
Non-Hodgkin’s lymphoma	9/9	100 (66–100)	0	-
Disseminated CMV infection	1/2	50 (9–91)	1/2	50 (9–91)
Invasive pneumococcal disease (<50 yo)	2/2	100 (29–100)	0	-
Pneumonia (<50 yo)	60/62	97 (88–100)	2/62	3 (0–12)
*P. jirovecii* pneumonia	0		3/3	100 (38–100)
Recurring pneumonia (>2 episodes/12 month)	5/5	100 (51–100)	0	-
Kaposi’s sarcoma	0		1/1	100 (17–100)
Syphilis	67/72	93 (84–97)	5/72	7 (3–16)
Mononucleosis-like syndrome	93/101	92 (85–96)	8/101	8 (4–15)

## Data Availability

Data available on request due to privacy.
